# Parental Online Information Access and Childhood Vaccination Decisions in North America: Scoping Review

**DOI:** 10.2196/20002

**Published:** 2020-10-13

**Authors:** Sarah Ashfield, Lorie Donelle

**Affiliations:** 1 Western University London, ON Canada

**Keywords:** childhood vaccination, parental vaccine decisions, online vaccine information, social media, vaccine hesitancy, digital health literacy

## Abstract

**Background:**

Immunizing children throughout their early years prevents the spread of communicable disease and decreases the morbidity and mortality associated with many vaccine-preventable diseases. Searching online allows individuals rapid access to health information.

**Objective:**

The purpose of this review was to develop an understanding of the existing literature of parents’ online health information-seeking behaviors to inform their vaccination choices for their children and to identify gaps in the literature around parents’ use of online health information and their vaccination choices.

**Methods:**

A scoping review of peer-reviewed literature from Canada and the United States was performed. The following databases were utilized to perform the search: PubMed, CINAHL, Nursing & Allied Health Database, Scopus, and PsycINFO. The purpose of this review was to examine parents’ use of online information seeking related to vaccine information and to understand how parents utilize this information to inform decisions about vaccinating their children. Of the 34 papers included in the review, 4 relevant themes and subthemes were identified: information seeking, online information resources, online vaccine content, and trust in health care providers.

**Results:**

Examination of the literature revealed conflicting information regarding parents’ use of social media and online resources to inform decisions around vaccinating their children. There is evidence of significant misinformation regarding vaccine risks online. Parents’ digital health literacy levels are unknown and may affect their ability to appraise online vaccination information.

**Conclusions:**

Parents are seeking vaccine information from online sources. However, the influence of online vaccine information on parental vaccine practices remains uncertain.

## Introduction

Vaccination programs are a vital contribution to public health practice in North America [1,2]. Immunizing children throughout their early years prevents the spread of communicable disease and decreases the morbidity and mortality associated with many vaccine-preventable diseases. Sustaining vaccination rates above 95% maintains community immunity and prevents outbreaks of vaccine-preventable diseases [3]. Despite ongoing public health efforts in North America, childhood vaccination rates are not meeting the established goals for effective disease prevention [4,5]. In 2019, outbreaks of measles, a highly contagious, vaccine-preventable disease, occurred in both the United States and Canada [6]. Health Canada has highlighted the importance of understanding factors associated with under-vaccinated and unvaccinated children, as well as vaccine hesitancy among parents and guardians, as key to improving vaccination rates in Canada [4]. Public health officials in the United States have also identified that parental delay and refusal of vaccinations threaten community immunity and identify this issue as a significant research priority [7]. The health implications for under-vaccinated children are concerning as the prevalence of communicable disease outbreaks become increasingly common. The use of digital information, including the prevalence of social media use among Canadian parents, highlights a need to understand the impact of online information on parents’ vaccine choices.

National immunization targets for Canadian children aim for 95% vaccination coverage by a child’s seventh birthday for the following diseases: diphtheria, tetanus, pertussis, polio, measles, mumps, and rubella [8]. Recently reported statistics demonstrate that vaccination rates fall short of the established immunization targets across Canada; immunization rates for 7-year-olds are 71% for diphtheria, tetanus, and pertussis; 90% for polio; and 86% for measles, mumps, and rubella [8]. American statistics also demonstrate immunization rates lower than 95%, with 83.2% of children aged 35 months having received at least 4 doses of diphtheria, tetanus, and pertussis vaccine; 92.7% having at least 3 doses of polio vaccine; and 91.5% having at least 1 dose of the measles, mumps, and rubella vaccine [9].

Vaccine hesitancy among parents or guardians is a growing public health issue reflected by the increased number of medical and nonmedical vaccine exemptions in both Canada and the United States [10]. Vaccine hesitancy is defined by the World Health Organization as the reluctance or refusal to vaccinate despite the availability of vaccines [11]. Despite continued efforts to improve childhood vaccination rates, both Canada and the United States are not meeting national goals, and nonmedical exemptions continue to proliferate [4,9].

Accessing information about the benefits and risks of childhood vaccines helps parents make informed decisions regarding vaccinating their children. Searching online via the internet allows individuals rapid access to health information. A 2016 survey of Canadians’ online activity demonstrated that 96% of Canadians aged 15-34 years and 93% of those aged 35-44 years use the internet on a daily basis [12], and as many as 79% of American internet users have searched online for health information [13]. While accessing health information online is important, digital health literacy or having the skills to seek, find, understand, and appraise online health information and then apply that knowledge to making an informed decision is critical [14]. While population-based assessments of Canadian digital health literacy levels are unknown, the health literacy of Canadians is concerning. Over 60% of Canadians have low health literacy skills that place them at higher risk of poor health [15,16]. Canadian adults’ health literacy skills were measured utilizing the International Adult Literacy and Skills Survey that assesses prose literacy, document literacy, numeracy, and problem-solving skills in different languages and cultures focused on broadly defined health content in the following areas: health promotion, health protection and accident prevention, disease prevention, and health care activities [16].

Vaccination information is available to most Canadians and Americans, although understanding and applying this information to ones’ individual health can be challenging. Online digital health information has evolved from static information retrievable from online websites to include interactive and collaborative sites where there is no central authority [17]. Within the context of childhood immunization information, parents are able to retrieve information but also contribute their personal knowledge and experience through interactive online social media platforms. Given the high prevalence of online information seeking among parents [18-20], investigating what information exists online related to childhood vaccinations and how parents use this information may provide insight into vaccination decision making. This scoping review examined research regarding parents’ use of online resources regarding primary schedule vaccinations, to understand where parents are searching online and how they utilize online information to inform vaccination choices for their infants and children.

A scoping review was undertaken for 2 reasons: to grasp an understanding of the existing literature of parents’ online health information-seeking behavior to inform vaccination choices for their children and to identify gaps in the literature around parents’ use of online health information resources and their vaccination choices.

The following research questions informed this review: “What are parents’ online information-seeking patterns and behaviors related to childhood primary immunization series?” and “How did parents use online resources to inform their decision regarding vaccination of their children?”

## Methods

A scoping review of the research literature was an appropriate method to examine this issue that is inclusive of qualitative, quantitative, and mixed methods literature to achieve a breadth of knowledge in this subject area. Researchers followed the collective guidelines of Colquhoun et al [21], Arksey and O’Malley [22], and Levac et al [23] to conduct this scoping review study. The steps involved in this review were identification of the research question; identification of relevant studies; study selection; charting the data; and collating, summarizing, and reporting the results [22].

### Identification and Study Selection

The following databases were searched: PubMed, CINAHL, Nursing & Allied Health Database, Scopus, and PsycINFO. The following search terms were used: vaccine, vaccines, vaccination, immunization, vaccinated, vaccinate, vaccine hesitancy, parent, parents, mother, mothers, father, fathers, parental, social media, digital health information, facebook, twitter, pinterest, snapchat, tumblr, Instagram, linkedin, google plus, youtube, reddit, flickr, vine, quora, periscope, whatsapp, and internet. Search terms were combined using AND or OR in the database search. A research librarian was consulted to assist with the search strategy. A justification search was completed with the Allied and Alternative Medicine (AMED) database; this search revealed no further articles in the subject area. Grey literature was located by searching Proquest Dissertations & Theses Global. Reference lists of articles were hand searched to identify any further literature that met the inclusion criteria.

#### Inclusion Criteria

Articles published from January 1, 2010 to December 31, 2019 in the English language from Canada and the United States were included. The aim was to focus on the past decade to reflect changes in information seeking that have occurred with widespread internet access in North America. The selected literature focused solely on the primary immunization series of children, parental decision relating to childhood vaccinations, online vaccination information seeking, and social media and childhood vaccinations.

#### Exclusion Criteria

**Figure 1:**
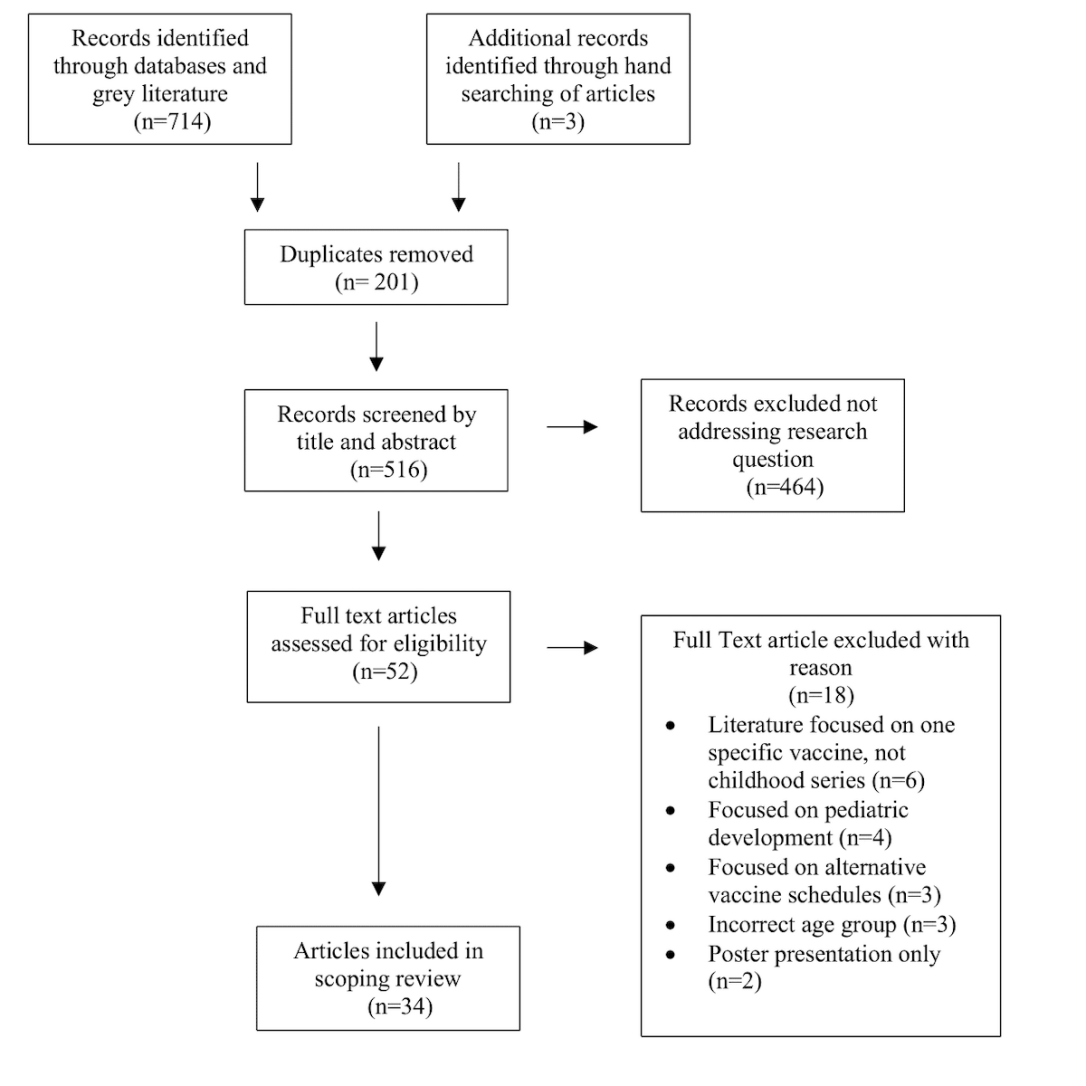
PRISMA process to select articles.

Articles published prior to January 1, 2010 were excluded as it is the authors’ intent to determine the use of current social media and internet. Articles that focused on vaccination outside of the primary childhood series such as human papillomavirus and influenza and adult and adolescent hepatitis vaccinations were excluded. Any articles that focused on general childhood development and on adolescent vaccination decision making were excluded, as the intention is to focus the topic on parents’ choices regarding vaccinations. Literature involving children who were able to consent to their own vaccines was also excluded from this review. Articles published in languages other than English were excluded, and articles where research was conducted outside of North America were excluded. Figure 1 illustrates the PRISMA selection process [24].

### Analysis

A title review of all articles was conducted for subject eligibility, followed by an abstract review and finally a full article review. A second reviewer read 15% of the articles to ensure consistency in data analysis to address the research question and purpose [22,23]. Discrepancies between inclusion of articles were discussed between the authors until consensus was reached. After eligible articles were identified, each was read several times for detail and to identify relevant categories and themes. Articles were identified by a numbering system and collated in a spreadsheet identifying country of origin, methodologies, limitations, instruments, methods, and key findings from an iterative and inductive analysis of all articles. Thematic analysis allowed for identification of relevant themes grounded in the data and gaps in the literature. An iterative analysis process was utilized by the authors; themes were identified through discussion and immersion in the literature. Discrepancies were reconciled through discussion until all themes were mutually agreed upon.

## Results

The eligibility criteria for inclusion in the study were met by 34 articles. The majority (28/34, 82%) of the literature is from the United States, and 18% (6/34) is from Canada. The majority (20/34, 58%) of the literature was qualitative in nature, 32% (11/34) was quantitative, and 9% (3/34) used mixed method studies. Of the mixed method studies, 2 studies were concurrent, and 1 was sequential. Literature on social media was focused on the second half of the decade, with all but one article published between 2015 and 2019. The following 4 broad themes were identified: information seeking, online information resources, online vaccination information content, and trust in health care providers. A narrative review of these themes follows.

### Information Seeking

One of the most prevalent themes identified throughout the literature was one of information seeking; 50% (17/34) of articles discussed this theme [18,19,25-39]. Parents are looking online for vaccination information [18,26,27,30,31,39].

Online information seeking may have implications for the way that parents perceive the health and safety consequences of childhood vaccinations [20]. Canadian researchers investigated the impact of parental online information seeking through surveying parents and found that parents who searched the internet for childhood vaccine information (in 2011 and 2014) are more likely to perceive vaccines as less safe than those who did not search on the internet [20]. Yet, in another study also performed in 2011 that surveyed American parents, researchers found that 95% of parents of school-aged children who chose not to have their children vaccinated listed their health care provider as their main source of information, with only 34.5% reporting the internet as a source of information [25].

Mossey et al [34] evaluated Canadian parents’ experiences in making vaccination decisions. Parents identified that searching for information was an important part of their decision-making process and that locating and interpreting online information was difficult at times [34]. Reportedly, some parents looked for information online to confirm information provided by their health care provider; parents expressed concerns about their health care provider’s lack of knowledge regarding childhood vaccines [35]. There is some evidence that parents who delayed or declined vaccinating their children specifically sought information through online social media platforms, such as blogs and videos, appreciating the personal experiences from other parents found on theses platforms [34]. Jones et al [18] examined the information sources of parents of school-aged children in the United States who refused at least one vaccine for their child(ren). Researchers assessed the impact of diverse information sources on vaccine attitudes, beliefs, and medical exemptions. In this study, 40% of all respondents reported that the internet was a good source of vaccine information; however, only 19.9% of all parents in this study reported using the internet as a source of vaccine information [18]. Those who reported using the internet to obtain information about vaccines were less likely to agree that their children needed or would benefit from vaccines and were more likely to have obtained a nonmedical vaccine exemption for their child [18].

Hwang and Shah [33] examined the associations between health information sources, parental perceptions of vaccine benefits, and maintenance of vaccine schedules. They evaluated magazines, newspapers, television, the internet (search engines, general websites, drug company websites, and other health websites), interpersonal communication (doctors, nurses or physician assistants, pharmacists, and friends), and social media (social networking sites, blogs or vlogs) as health information sources [33]. Parents that valued social media as a source of health information reported fewer perceived benefits (identified as vaccine benefits outweighing vaccine risks) of vaccinations [33]. Positive parental perceptions of vaccine benefits were strongly associated with the maintenance of vaccination schedules [33].

Berreth [25] assessed sources of information among parents of school-aged children in the United States. They found that 95.4% of parents sought vaccine advice from their health care provider, 51.1% from family and friends, 34.5% from the internet, 25.3% from the news, and 15.5% from television and radio. Parents who cited the internet as a source of information listed the sites they most frequently used as webmd.com (34.4%), mayoclinic.com (27.5%), and government or other medically endorsed sites (68.9%) [25]. Researchers compared information sources of parents who vaccinated their child with those who exempted their children from vaccines. While 83.3% of immunizers versus 77.8% of vaccine abstainers identified health care providers as sources of vaccine information, only 25% of parents who immunized their children utilized the internet as a source of information compared to 44.4% of parents who did not immunize [25]. Deas et al [29] interviewed parents of school-aged children in 3 counties in the US state of Oregon with low immunization rates (combined pediatric immunization rate of 65%). Researchers found that all parents, regardless of vaccine acceptance or hesitancy, dismissed social media as they found it an unreliable source of vaccine information [29].

However, when exposed to evidence-informed online information, parental attitudes regarding childhood vaccinations shifted; researchers found that, after exposure to a vaccine information website developed by experts in the field, vaccine-hesitant parents had a more positive attitude toward vaccines compared to vaccine-hesitant parents with no exposure to the evidence-informed website [28]. Glanz et al [32] evaluated vaccine information-seeking behaviors in parents who accessed information from an online website. The website was developed and mediated by several experts (pediatrician, vaccine safety researcher, and risk communication specialist). Parents in this study preferred to engage with online experts about vaccinations rather than interacting with other parents within this online site [32].

Researchers who focused on the composition of search terms used by parents seeking online vaccine information found that parents who utilized positive search terms (eg, “vaccine benefits”) when looking for childhood vaccine information encountered few myths about vaccine safety and effectiveness [40]. In contrast, parents who used negative search terms (eg, “vaccine risks”) found 4.8 times more misinformation or myths than a person using positive terminology [40]. The nature of the search terms used to find online vaccination information can alter the results and the information made available to parents [40].

### Online Information Resources

Parents searched online for vaccine information using common search engines such as Google and Yahoo, and many used popular social media sites such as Facebook, Instagram, Pinterest, Twitter, and YouTube [27,38,41-43]. One study found that the internet was listed within the top 3 most common sources for vaccine information among those who vaccinated their children, and 46% of parents who used the internet reported using search engines such as Google and Yahoo to search online for vaccine information [27].

The popularity of social media has given rise to prosumers — individuals who not only access online information but produce online content. Researchers found that websites that allow users to post online content without verification of information promoted antivaccination messages through antigovernment views, celebrities, personal stories, and naturalist arguments [43]. A Canadian assessment of online vaccine information websites (identified through searching Google, Facebook, Twitter, and YouTube) targeted to parents found that the majority of websites offered poor-quality information regarding childhood vaccination [42]. Researchers rated the websites with a communication index tool developed by the Centers for Disease Control and reported that 5% of materials (3 websites) met the standards for clear communication [42]. However, websites that monitored user-generated content and required academic references demonstrated a balance between openness and credibility [43].

### Online Vaccination Information Content

Researchers assessed the content of online vaccine information, and there is evidence that content was conflicting and inconsistent [20,41,43-47].

Regarding online websites, American researchers investigating online information content generated from Google searching found that 59% of the first 100 Google sources were provaccine and 41% were antivaccine [43]. Similarly, Kata [46] sought antivaccination websites to assess their content and accuracy. Researchers utilized neutral vaccine information-seeking search terms (“vaccine,” “vaccination,” and “immunization OR immunisations”) to search American and Canadian versions of Google and found that, within the first 10 results, 24% of American and 13% of Canadian results were antivaccine [46]. A content analysis revealed that all of the assessed antivaccine websites claimed that vaccines are poisonous and cause idiopathic illnesses [46]. As well, 88% of the websites contained information that challenged the evidence about the efficacy of vaccines and whether vaccines conferred immunity, 88% endorsed alternative treatments (homeopathy, chiropractic care, naturopathy, or acupuncture) as superior to vaccination, 75% of the websites made accusations that regulatory bodies have information about vaccines that they are hiding from the public, and 75% of the websites suggested that vaccine promotion is solely motivated by profit seeking [46].

The content developers of websites advocating antivaccine messages used sophisticated strategies to communicate their perspective [48,49]. Researchers examined the content of antivaccine websites and found that these websites used persuasive tactics, attacked the credibility of vaccine advocates, expressed mistrust about scientific evidence, and used psuedoscientifc evidence to support claims in favor of antivaccination [49]. Similarly, Getman et al [48] performed a network analysis of over 50,000 websites that contained vaccine-relevant content to determine the structure and influence of the online vaccine-hesitant community. They discovered effective use of hierarchical scientific language by the vaccine-hesitant community to enforce their online authority [48].

Regarding social media vaccine resources, researchers evaluated literature on vaccine information on the following social media platforms: YouTube, Twitter, Pinterest, Facebook, and various blogs. Facebook was identified as a vaccine information resource for parents in several studies [20,38,42,50]. The majority of research literature that focused on social media use among parents was published between 2015 and 2019, reflecting parents’ recent use of social media for seeking vaccine information. Parents also accessed social media sites to understand other parents’ experiences with vaccination processes; peer-to-peer information was a valued source of information accessible on social media sites [50]. Across social media platforms, there was a mix of positive and negative vaccine messaging. Our findings indicate widespread mistrust of government institutions and skepticism towards the vaccine industry across all social media platforms except for pediatrician-authored blogs [37,41,44,45,47,50-53]. Vaccine-hesitant online communities tend to leverage scientific and academic language to enforce their antivaccination narrative [48]. Consistent messaging about the dangers of vaccines was present on Twitter, Facebook, YouTube, parenting blogs, and Pinterest. The dominant message across social media was one of elevated health risks among vaccinated children — perceived as information concealed by government and industry.

### Trust in Health Care Providers

The concept of trusted health care providers was prevalent in many of the reviewed studies [18,20,27,30,31,34,35,45, 46,49,50]. Parents often listed their physician or health care provider as a trusted source of vaccination information [18,27,30,31]. Eller et al [30] examined the association between the level of trust a mother had with their pediatrician and vaccine information sources. Mothers who trusted their pediatrician were 2.47 times more likely to list their pediatrician as their main source of vaccine information compared to mothers who had not established a trusting relationship with their pediatrician [30]. Parents who chose to vaccinate their children reported their physician as the biggest influence in their decision to vaccinate [27].

However, parents who identified as vaccine-hesitant or those who did not vaccinate their children were less trusting of information conveyed by their health care provider [27]. Parents who chose to delay or declined vaccinating their children tended to seek information from a diverse group of health disciplines such as cardiologists, health researchers, health care students, and homeopathic practitioners [34]. Kata [46] identified mistrust in the medical system as a strong message on antivaccine websites and on an antivaccination Facebook group [46,50].

## Discussion

This scoping review of 34 articles investigated parents’ use of online information to inform vaccination choices for their children. Overall, research studies were broadly focused on understanding the content of online vaccine information and parents’ online information seeking to inform their vaccine decisions. Our analysis of the current literature indicates that parents are actively looking online for vaccination information. Vaccination information was found on the social media platforms of YouTube, Twitter, Pinterest, and Facebook as well as various types of blogs. However, there is conflicting information within the published research regarding parental trust in the information found online and utilization of online information. Google was reported as the main online search engine among parents seeking vaccine information in both countries. The search terms that parents used in their online information seeking significantly impacted the disposition of the vaccine information and exposure to the number of vaccine myths retrieved online. Trust in health care providers plays an important role in information seeking. Parents who trust their health care provider tend to value them as an accurate source of vaccine information while those who do not trust their health care provider often seek information online. This is consistent with literature that demonstrates higher quality of life, more beneficial health behaviors, and higher treatment satisfaction when patients trust their health care provider [54].

The challenge for parents seeking vaccine information was the conflicting information found online. The influence of online vaccine information on parental vaccination practices (provaccination vs antivaccination) remains uncertain. A continuum of online information seeking among parents ranged from a complete dismissal of online information to regular online information access to inform their vaccine decision making. Due to the limited amount of Canadian research available, direct comparison between Canada and the United States was not plausible. Existing Canadian research findings highlight the relationship between online information seeking and parental antivaccination sentiment; parents seeking information online were more likely to perceive vaccines as less safe and be less inclined to adhere to the recommended vaccine schedule [20]. However, a greater number of studies in this area is needed to substantiate these findings. There is a need for further research performed in Canada on parents’ use of online vaccine information to determine how it informs their vaccine decisions.

Essentially absent in the published research was an understanding of parents’ digital health literacy skills. Only one of the studies in this review considered parents’ digital health literacy. The ability to interact with health information becomes more complex within the digital health context. Digital health literacy refers to the knowledge and skills inclusive of the ability to read and understand general information (traditional literacy and numeracy); effectively use digital devices, which includes awareness of data privacy, security, and ownership (computer literacy); critically understand and assess sophisticated media messages (media literacy); discern what is reliable and valid health information (scientific literacy); source information (information literacy); and navigate the health care system (health literacy) [14]. Although parents are looking online for information to inform their decision, the impact of parents’ health literacy and digital health literacy skills that support access to credible information sources and an ability to critically assess the vaccination information is a significant gap in the research literature [55,56]. In fact, research exploring parents’ information ecosystems is warranted to fully understand their information-seeking practices, preferred resources, and ability to critically evaluate vaccination-related information.

There was no research in this review that focused on parents’ use of Instagram as a source for vaccine information. Research focused on parental use of Instagram and other emerging popular social media networks as a source for vaccine information is warranted.

The influence of misinformation on parental vaccine choices is an issue that may have significant implications for maintaining community immunity. Parents encounter inaccurate and false vaccine information, vaccine conspiracy theories, and vaccine myths propagated online, especially on social media sites [42,52]. Parents, in their search for information, may be exposed to persuasive tactics that perpetuate myths and fear mongering. Persuasive tactics combined with misinformation and myths may cause parents to believe that vaccines themselves are a threat to their child’s health. Similar findings on the harms of social media rumors and misinformation surrounding COVID-19 also demonstrate the detrimental effects of online myth propagation. Improper use of pharmacological drugs and panic buying have resulted from online COVID-19 myths [57], as well as concern over the disease being spread through meat consumption and Chinese biological military laboratories [58].

Given the diversity of health information sources, health care providers along with public health organizations need to work together with popular social media platforms to ensure information accuracy. Recently, some social media platforms have implemented measures to prevent the propagation of antivaccine messaging and misinformation. Twitter recently (2019) integrated a search tool into their platform that directs users to a sanctioned US government vaccine website [59]. Pinterest also implemented community guidelines in 2019 that limit misinformation; Pinterest now removes pins that promote antivaccine advice and redirects individuals to reputable vaccine information sources such as the World Health Organization [60]. Facebook and Instagram also implemented similar policies in 2019. Facebook suggests users visit a public health website for vaccine information, and Instagram blocks some false information posts and reports using third-party fact checkers to help reduce false information [61]. However, the prevalence of online vaccination myths including the misconception that vaccination causes autism persists [18,20,53], demonstrating that further work needs to be done to dispel unsubstantiated vaccine myths.

Given the prevalence of online antivaccine information and the use of hierarchical and authoritative language among the online vaccine-hesitant community to promote the antivaccine sentiment, [48] the composition of information-seeking factors has the potential to influence the nature of information that parents are accessing. Algorithms are used by online search engines such as Google to tailor information and influence the outcome of individuals’ online information seeking [62]. The algorithms determine the information outcome based on factors such as the search terms used, country, and type of digital device [62]. One of the algorithms used by Google determines if content is reliable and demonstrates “expertise, authoritativeness, and trustworthiness” on a given topic [62]. Ruiz and Bell [40] determined that the character or disposition of a Google search for vaccine information significantly affects the outcome of the search. Parents who are concerned about and search for risk information regarding vaccines will encounter more vaccine myths than parents who use neutral search terms to search for vaccine benefits [48]. Understanding that Google is the most popular online search engine, health care providers may want to consult with parents regarding their search terms for online vaccination-related information. Further research exploring the impact of search algorithms and information-seeking behaviors regarding online vaccination information would be beneficial. It may be that clinicians not only “prescribe” evidence-informed online resources to parents but in addition will need to consider the composition of search strategies (eg, search terms or strings) to mitigate parents’ access to vaccine misinformation and myths. The use of information prescriptions has been successful in the past for online searching in the pediatric parent population [63]. Educating parents about the benefits and risks of searching online and prescribing search terms may allow parents to access evidence-informed information to facilitate informed decision making and also mitigate the potential harms of online vaccine myths. Research on the use of a search term prescription in this population is required.

### Limitations

This review evaluated parental information seeking in those who have internet access; it did not capture parents who have limited or no access to online health information. The online environment is fluid due to its interactive user-driven features with websites and social media evolving and changing from day to day. Findings involving online information should be viewed cautiously as digital information changes rapidly. This review included English-only research reports, and research literature in languages other than English may have findings that are different than reported here.

### Conclusion and Implications

This review identified that parents are looking online on major search engines and social media platforms for vaccine information. It was identified that locating accurate information online regarding the benefits and risks of vaccines is challenging for parents given the low number of sources that contain accurate information [42]. There was conflicting evidence about how parents utilize information found online to inform their vaccine choices. However, vaccine-hesitant parents who have access to accurate online vaccine information have significant improvement in attitudes regarding vaccination benefits and reduction in parental concerns about vaccination risks [28]. Given the plethora of misinformation perpetuated online, clinicians may want to provide “information prescriptions” to parents regarding the search terms they use and encourage parents to access websites moderated by health care experts. The interactive component to the websites would provide an opportunity for parents to ask questions of vaccination experts. Health care providers should discuss with parents the nature of online vaccine discussions. Reviewing with parents the utilization of hierarchical and scientific language utilized by some to promote antivaccine messaging. Further discussion focused on search terms and even providing parents suggestions for vaccine-positive or neutral terminology that will allow them access to a more balanced discussion of vaccine benefits and risks online.

Parents identify trust as a fundamental part of the vaccine decision-making process. This importance placed upon perceived trust in the source of information reinforces the importance of relational care practices, and a trusting relationship with a health care provider is a priority. Developing and fostering trust between primary care providers and parents may be a strategy to increase vaccine uptake by parents.
